# CtBP1 associates metabolic syndrome and breast carcinogenesis targeting multiple miRNAs

**DOI:** 10.18632/oncotarget.7711

**Published:** 2016-02-25

**Authors:** Paola De Luca, Guillermo N. Dalton, Georgina D. Scalise, Cristian P. Moiola, Juliana Porretti, Cintia Massillo, Edith Kordon, Kevin Gardner, Florencia Zalazar, Carolina Flumian, Laura Todaro, Elba S. Vazquez, Roberto Meiss, Adriana De Siervi

**Affiliations:** ^1^ Laboratorio de Oncología Molecular y Nuevos Blancos Terapéuticos, Instituto de Biología y Medicina Experimental (IBYME), CONICET, Buenos Aires, Argentina; ^2^ Departamento de Fisiología, Biología Molecular y Celular, Facultad de Ciencias Exactas y Naturales (FCEN), Universidad de Buenos Aires (UBA), and Instituto de Fisiología, Biología Molecular y Neurociencias (IFIBYNE), CONICET, Buenos Aires, Argentina; ^3^ National Cancer Institute and National Institute of Minority Health and Disparities, National Institutes of Health, Bethesda, MD, USA; ^4^ Área de Investigación del Instituto de Oncología A.H. Roffo, Universidad de Buenos Aires, Buenos Aires, Argentina; ^5^ Laboratorio de Inflamación y Cáncer, Departamento de Química Biológica, Facultad de Ciencias Exactas y Naturales (FCEN), Universidad de Buenos Aires (UBA), IQUIBICEN – CONICET, Buenos Aires, Argentina; ^6^ Departamento de Patología, Instituto de Estudios Oncológicos, Academia Nacional de Medicina, Buenos Aires, Argentina

**Keywords:** CtBP1, metabolic syndrome, high fat diet, breast cancer, miRNAs

## Abstract

Metabolic syndrome (MeS) has been identified as a risk factor for breast cancer. C-terminal binding protein 1 (CtBP1) is a co-repressor of tumor suppressor genes that is activated by low NAD+/NADH ratio. High fat diet (HFD) increases intracellular NADH. We investigated the effect of CtBP1 hyperactivation by HFD intake on mouse breast carcinogenesis. We generated a MeS-like disease in female mice by chronically feeding animals with HFD. MeS increased postnatal mammary gland development and generated prominent duct patterns with markedly increased CtBP1 and Cyclin D1 expression. CtBP1 induced breast cancer cells proliferation. Serum from animals with MeS enriched the stem-like/progenitor cell population from breast cancer cells. CtBP1 increased breast tumor growth in MeS mice modulating multiple genes and miRNA expression implicated in cell proliferation, progenitor cells phenotype, epithelial to mesenchymal transition, mammary development and cell communication in the xenografts. These results define a novel function for CtBP1 in breast carcinogenesis.

## INTRODUCTION

Breast cancer is the leading cause of cancer death among women, after skin cancers [1]. Although genetic susceptibility influences cancer risk, non-inherited factors determine most of the differences in cancer risk across populations and among individuals [2, 3]. It is estimated that around 30% of total cancer deaths in United States could be attributed to life style, diet and physical activity; all factors associated to metabolic syndrome (MeS) [4].

Based on the National Cholesterol Education Program's Adult Treatment Panel III (NCEP ATP III) criteria, MeS is a cluster of pathophysiological disorders that comprises at least three of the following factors: abdominal obesity (waist circumference ≥ 35 inches in women), triglycerides ≥150 mg/dL, high density lipoprotein cholesterol (HDL-C) < 50 mg/dL in women, blood pressure ≥ 130/85 mmHg, and fasting glucose ≥ 110 mg/dL [5].

Several studies have established that components of MeS are positively correlated with breast cancer development [6–9]. In addition, MeS has been associated with breast cancer risk in women, and this correlation is stronger within postmenopausal population [10–12].

Recently, we have reported that gene transcription regulation by C-terminal binding protein 1 (CtBP1) provides a molecular link among MeS, CtBP1 function and tumor growth in prostate cancer [13]. CtBP1 is a transcriptional co-repressor of tumor suppressor genes, such as BRCA1, PERP, PTEN, p21^CIP1/WAF1^, Bax, Noxa and E-cadherin [14]. CtBP1 was proposed as a metabolic cellular sensor [15] since its transcriptional regulatory activity is differentially modulated by the nuclear NAD+/NADH ratio, showing much higher affinity (>100-fold) for NADH compared with NAD+ [16].

There are numerous *in vitro* studies supporting that CtBP1 regulates multiple genes related to tumorigenesis, tumor progression and metastasis in breast cancer cells [17–22]. Clinical studies demonstrated that CtBP1 overexpression was observed in invasive ductal breast cancer compared to normal breast tissue [18]. In addition, CtBP1 protein expression in breast cancer patients is associated with lower median survival [17]. However, the effect of CtBP1 hyperactivation by MeS in breast cancer development and progression remains unexplored.

In this work we examined the role of CtBP1 in breast carcinogenesis and tumor growth using a MeS experimental model. We found that MeS increased mammary gland development and induced CtBP1 expression in mammary ducts. MeS was also associated with the expansion of the stem/progenitor-like cell population. CtBP1 expression induced breast cancer cells proliferation by inhibiting cell cycle arrest. Importantly, CtBP1 increased breast tumor growth in our preclinical orthotopic xenograft model regulating the expression of mRNAs and miRNAs involved in cell proliferation, cell communication, progenitor cells phenotype, epithelial to mesenchymal transition (EMT) and mammary development in breast cancer tumors.

## RESULTS

### HFD induced MeS in female athymic *nude* mice

**Figure 1:**
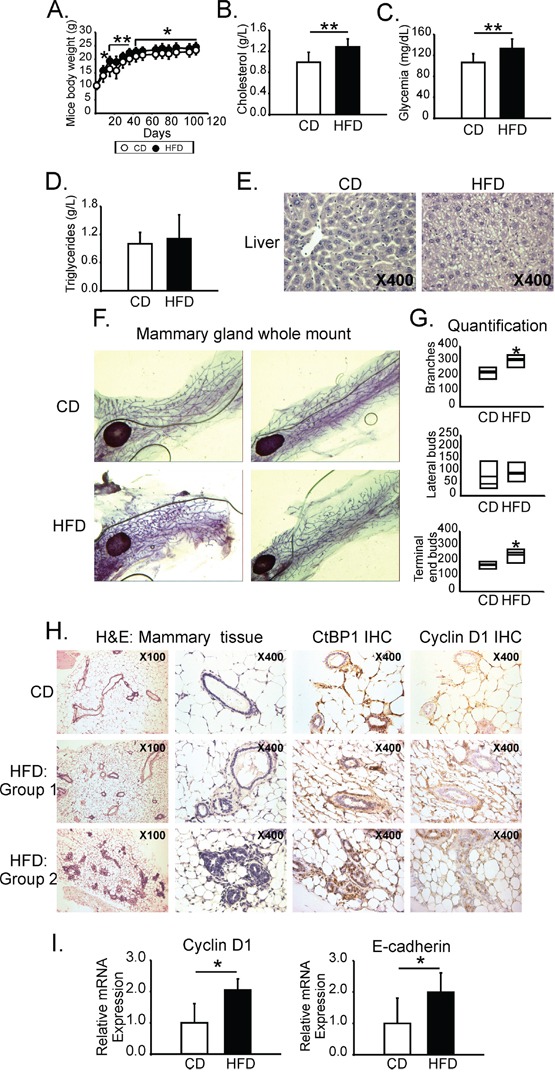
MeS increased postnatal mammary gland development and induced CtBP1 expression in mammary ducts Female *nu/nu* mice (N=24) were chronically fed (16 weeks) with HFD or CD. **A.** Mice body weight follow up during the experiment. **B.** Cholesterol, **C.** Glucose, and **D.** Triglycerides serum levels determined after mice euthanasia. Histograms show media and SD values of one representative experiment from two biological replicates. Significance was analyzed by t-test (*, p < 0.05; **, p < 0.01). **E.** H&E staining from liver from animals fed with CD or HFD. Magnifications x400. **F.** Mammary glands from mice fed with HFD or CD were whole mounted and stained with aluminum carmine red solution. **G.** Branches, lateral buds and TEB from mammary ducts were quantified. Plots show media and SD values of one representative experiment (N=2) with seven replicates each (*, p value < 0.05). **H.** H&E staining and IHC using anti-CtBP1 and anti-Cyclin D1 specific antibodies were performed in mammary tissue from mice fed with CD or HFD. *HFD Group 1:* mammary glands from HFD fed mice that show normal ducts; *HFD Group 2:* mammary glands from HFD fed mice that show prominent duct pattern. Magnifications are x40. *I.* RNA from mammary tissue was isolated and Cyclin D1 and E-cadherin expression were determined by RT-qPCR. Data were normalized to β-actin and CD (*, p value < 0.05).

To analyze the involvement of CtBP1 in breast carcinogenesis, female *nu/nu* mice were chronically fed with HFD or CD. Body weight was measured weekly. Animals were sacrificed and biochemical parameters were determined at the endpoint. Consistent with a MeS-like disease, HFD fed group showed a significant increase in body weight (Figure 1A), serum hypercholesterolemia (Figure 1B) and hyperglycemia (Figure 1C) compared to control animals with no differences in the triglyceride levels (Figure 1D). Histopathological analysis revealed that HFD mice developed liver diffuse steatosis (Figure 1E) and kidney glomerular and tubular non-specific alterations (data not shown).

### MeS increased postnatal mammary gland development and induced CtBP1 expression in mammary ducts

Whole mount assay and histological analysis from mammary glands showed a significantly increased in the number of branches and TEB in MFP from HFD fed animals (Figure 1F, 1G). In addition, 44% of mammary ducts from HFD mice were covered with prominent epithelial cells with nuclear pseudostratification and columnar changes (Figure 1H-Group 2 H&E). None of the mammary glands from CD animals developed this feature. Interestingly, CtBP1 and Cyclin D1 expression positively correlated with these lesions (Figure 1H-Group 2). Consistently with an increase in mammary gland epithelial tissue, MeS induced Cyclin D1 and E-cadherin mRNA expression levels in the mammary tissue of experimental animals (Figure 1I).

### Serum from mice with MeS increased the stem-like/progenitor cell population in breast cancer cells

**Figure 2:**
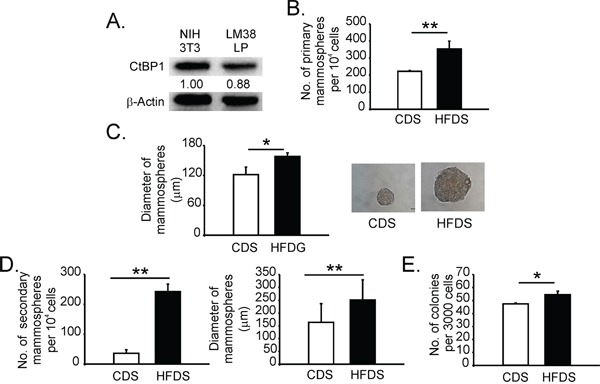
Serum from mice with MeS increased the stem-like/progenitor cell population in breast cancer cells **A.** CtBP1 expression was determined by WB in the indicated cell lines. Band quantifications performed by Image J software are indicated. Data were normalized to β-actin and NIH 3T3 cells. **B.** LM38-LP cells were exposed to CDS or HFDS during 6 days, and mammosphere formation assay was performed. Number of mammospheres was quantified. **C.** *Left panel:* Diameter of mammospheres (*, p value < 0.05). *Right panel:* mammospheres images. **D.** Mammospheres derived from LM38-LP were exposed to CDS or HFDS during 6 days and secondary mammosphere formation assay was performed. Number and diameter of mammosphere were determined. **E.** Clonogenic assay was performed in LM38-LP cells exposed to CDS or HFDS during 6 days. Histograms show media and SD values from three replicates (*, p value < 0.05; **, p value < 0.01). Magnifications x40.

To investigate MeS effects on stem-like/progenitor population of tumor cells, LM38-LP breast cancer cells with detectable CtBP1 expression (Figure 2A), were exposed to medium supplemented with serum obtained from HFD (HFDS) or CD fed mice (CDS) and a mammosphere formation assay was performed. We performed mammospheres using this particular cell line due to MDA-MB-231 does not form these features. Serum from mice with MeS augmented both the number and size of LM38-LP mammospheres (Figure 2B–2C). In addition, a secondary mammosphere formation assay was conducted exposing LM38-LP-derived primary mammospheres to HFDS or CDS. Both sera, CDS and HFDS, diminished the capability of the primary mammospheres to form secondary ones (Figure 2B *versus* D). Strikingly, mammospheres exposed to HFDS showed increased capability to form secondary spheres compared to CDS (Figure 2D). We also developed clonogenic assays by exposing LM38-LP breast cancer cells to both sera. HFDS increased colony formation compared to CDS (Figure 2E). In summary, our results demonstrate that HFDS enriched the progenitor population of breast cancer cells and increased their capability to proliferate.

### CtBP1 expression increased breast cancer cell proliferation inhibiting cell cycle arrest and inducing Cyclin D1 expression

**Figure 3:**
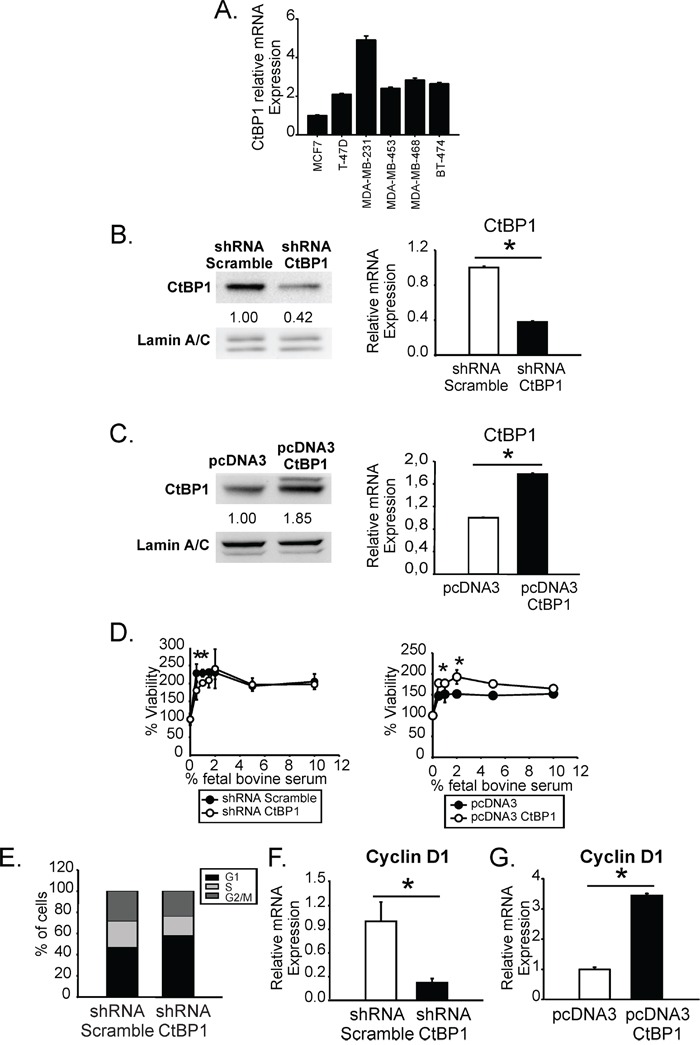
CtBP1 expression increased breast cancer cell proliferation inhibiting cell cycle arrest and inducing Cyclin D1 expression **A.** CtBP1 expression was determined in MCF7, T-47D, MDA-MB-231, MDA-MB-453, MDA-MB-468 and BT-474 cells by RT-qPCR. **B.** CtBP1 expression was determined in CtBP1-diminished expression stable transfected MDA-MB-231 cells and control cells by WB (*left*) and RT-qPCR (*right*). Numbers below bands indicate quantification using Image J software. Data were normalized to lamin A/C for WB or β-actin for RT-qPCR and control cells (*, p value < 0.05). **C.** CtBP1 expression was determined by WB and RT-qPCR as indicated above in MDA-MB-231 transiently transfected with CtBP1 overexpression or control vector. **D.** Stable (*left*) or transient (*right*) transfected MDA-MB-231 cells were exposed to the indicated percentages of FBS during 72 h and cell viability was determined by MTS assay. Media and SD values of one representative experiment (N=2) from three replicates are shown (*, p value < 0.05). **E.** Stable transfected MDA-MB-231 cells were grown with media without FBS during 72 h and cell cycle analysis was performed. Media and SD values from two biological replicates are shown. **F-G.** Cyclin D1 expression was determined in the indicated cells by RT-qPCR. Data were normalized to β-actin and control cells (*, p value < 0.05).

We analyzed CtBP1 expression levels in different breast cancer cell lines: MCF7, T-47D, MDA-MB-231, MDA-MB-453, MDA-MB-468 and BT-474. Triple negative breast cancer cells showed CtBP1 increased expression levels compared to luminals (Figure 3A). We continued our experiments using MDA-MB-231 cells with the highest CtBP1 expression levels (Figure 3A).

We generated CtBP1-depleted expression stable transfected cells and CtBP1-transiently overexpressing cells derived from MDA-MB-231 cells. Both, CtBP1 protein and mRNA levels were determined by WB and RT-qPCR, respectively (Figure 3B–3C). As shown, shRNA CtBP1 transfection decreased 60% of CtBP1 expression (Figure 3B); while pcDNA3 CtBP1 transfection induced 2 fold CtBP1 expression (Figure 3C).

We assessed proliferation of CtBP1 expression modulated cells growing at the FBS range 0 to 10%. We found that CtBP1 significantly increased MDA-MB-231 cell proliferation at low (< 2%) FBS concentration (Figure 3D). In addition, CtBP1 depletion induced an accumulation of cells in G1 phase comparing to controls, when cells were cultured without FBS (Figure 3E). Furthermore, CtBP1 depletion (Figure 3F) and overexpression (Figure 3G) significantly decreased or induced Cyclin D1 expression, compared to control cells.

Altogether, these results demonstrate that CtBP1 is implicated in cell proliferation since its depletion induces cell cycle arrest and inhibits cell proliferation in breast cancer cells.

### CtBP1 increased breast tumor growth in mice

**Figure 4:**
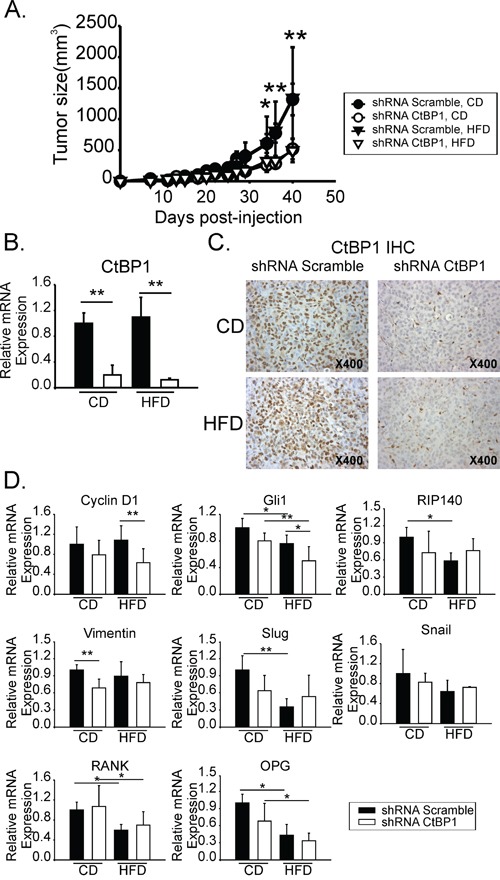
CtBP1 increased breast tumor growth in mice with MeS **A.** Tumor growth from CD or HFD orthotopic xenografts inoculated with shRNA scramble or shRNA CtBP1 cell lines. Curves indicate media and SD values of one representative experiment with 6 mice. **B.** CtBP1 RT-qPCR from HFD or CD xenograft samples. Data were normalized to β-actin and control. **C.** CtBP1 IHC from tumor xenografts. Magnification x400. **D.** RT-qPCR from xenograft tumors described above. Specific primers for the indicated genes were used. Data were normalized to β-actin and control. (*, p < 0.05; **, p < 0.01).

We inoculated CtBP1 depleted (shRNA CtBP1) or control (shRNA Scramble) MDA-MB-231 cells into the MFP from control or MeS *nu/nu* mice. Tumor size was monitored and after 41 days animals were sacrificed and tumor samples were collected for histological and RT-qPCR analysis. A significantly decreased tumor growth was observed in CtBP1 depleted xenografts compared to controls in both, HFD or CD fed mice (Figure 4A). As shown in Figure 4B and 4C, the diminution of CtBP1 expression levels was confirmed in tumors from each group.

### CtBP1 and MeS regulated the expression of genes that are involved in cell proliferation, progenitor cells phenotype, EMT and mammary development in breast tumors

Expression of genes involved in cell proliferation (Cyclin D1), progenitor cell phenotype (Gli1, RIP140), EMT (Vimentin, Slug, Snail) and mammary development (RANK, OPG, RIP140) were determined by RT-qPCR from CtBP1 depleted or control xenografts developed in MeS or control mice. We found that CtBP1 modulated expression of genes which conferred to tumors a highly proliferative, dedifferentiated and mesenchymal phenotype (Figure 4D). HFD significantly decreased Gli1, RIP140, Slug, RANK and OPG expression. CtBP1 depletion diminished Vimentin and Cyclin D1 expression. Interestingly, Cyclin D1 was the only tested target that was significantly regulated by CtBP1 in the MeS group.

Altogether these results suggest that Cyclin D1 is an important CtBP1 target modulated in the mammary gland and in breast tumor by MeS.

### CtBP1 modulated multiple miRNAs involved in metabolic process, cell cycle and cell communication in breast cancer associated to MeS

To determine miRNA expression profile associated to CtBP1 and MeS, GeneChip miRNA 4.0 Affymetrix was hybridized to total RNA isolated from CtBP1 depleted or control xenograft tumors generated in mice with MeS. After data normalization, we set the threshold at 1.5 fold induction for up- and −1.5 for down-regulated miRNAs. We identified 42 CtBP1 regulated miRNAs: 28 up- and 14 down-regulated (Table 1).

**Table 1:** List of differentially expressed miRNAs

miRNA_ID	Accession	shRNA Scramble/shRNA CtBP1	ANOVA p-value	FDR p-value
hsa-miR-4697-5p	MIMAT0019791	**4.32**	0.018	0.994
hsa-miR-664b-3p	MIMAT0022272	**3.24**	0.018	0.994
hsa-let-7e-3p	MIMAT0004485	**2.72**	0.015	0.994
hsa-miR-4448	MIMAT0018967	**2.71**	0.007	0.994
hsa-miR-223-3p	MIMAT0000280	**2.4**	0.015	0.994
hsa-miR-6885-5p	MIMAT0027670	**2.37**	0.031	0.994
hsa-miR-6721-5p	MIMAT0025852	**2.25**	0.035	0.994
hsa-miR-3151-5p	MIMAT0015024	**2.13**	0.045	0.994
hsa-miR-6746-3p	MIMAT0027393	**2.05**	0.006	0.994
hsa-miR-6770-3p	MIMAT0027441	**1.97**	0.014	0.994
hsa-miR-6743-3p	MIMAT0027388	**1.95**	0.008	0.994
hsa-miR-6080	MIMAT0023705	**1.87**	0.040	0.994
hsa-miR-6840-5p	MIMAT0027582	**1.78**	0.020	0.994
hsa-mir-4632	MI0017259	**1.76**	0.032	0.994
hsa-miR-3180	MIMAT0018178	**1.76**	0.035	0.994
hsa-miR-6781-5p	MIMAT0027462	**1.68**	0.042	0.994
hsa-miR-146a-5p	MIMAT0000449	**1.66**	0.032	0.994
hsa-miR-4442	MIMAT0018960	**1.65**	0.006	0.994
hsa-miR-1231	MIMAT0005586	**1.61**	0.017	0.994
hsa-miR-6881-5p	MIMAT0027662	**1.61**	0.043	0.994
hsa-miR-3180-3p	MIMAT0015058	**1.61**	0.043	0.994
ENSG00000199370	ENSG00000199370	**1.59**	0.047	0.994
hsa-miR-6863	MIMAT0027627	**1.57**	0.034	0.994
hsa-miR-637	MIMAT0003307	**1.55**	0.005	0.994
hsa-miR-4750-5p	MIMAT0019887	**1.55**	0.047	0.994
U101	U101	**1.54**	0.045	0.994
hsa-miR-4302	MIMAT0016855	**1.52**	0.041	0.994
hsa-miR-1271-3p	MIMAT0022712	**1.5**	0.047	0.994
hsa-mir-194-5p	MI0000488	**−1.5**	0.040	0.994
hsa-miR-1285-3p	MIMAT0005876	**−1.51**	0.048	0.994
hsa-miR-494-3p	MIMAT0002816	**−1.53**	0.012	0.994
hsa-mir-381	MI0000789	**−1.53**	0.037	0.994
hsa-mir-433	MI0001723	**−1.59**	0.032	0.994
hsa-mir-548n	MI0006399	**−1.59**	0.037	0.994
hsa-miR-6791-3p	MIMAT0027483	**−1.62**	0.048	0.994
hsa-mir-522	MI0003177	**−1.74**	0.033	0.994
ENSG00000201042	ENSG00000201042	**−1.76**	0.023	0.994
SNORA38B	SNORA38B	**−1.76**	0.023	0.994
hsa-miR-4793-5p	MIMAT0019965	**−1.77**	0.010	0.994
hsa-miR-6798-3p	MIMAT0027497	**−2.12**	0.007	0.994
hsa-miR-940	MIMAT0004983	**−2.48**	0.035	0.994
hsa-miR-378a-3p	MIMAT0000732	**−3.11**	0.044	0.994

**Figure 5:**
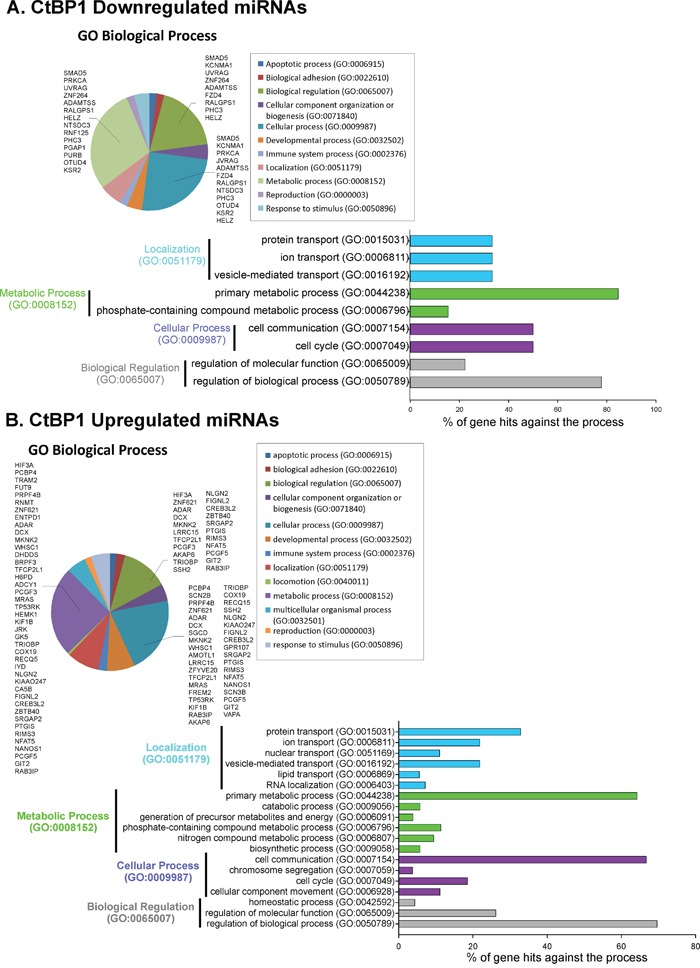
CtBP1 modulates miRNAs involved in metabolic process, proliferation and cell communication in breast cancer associated to MeS GeneChip miRNA 4.0 Affymetrix was hybridized to total RNA from xenografts with CtBP1 knockdown or control grown in mice fed with HFD. Predicted target genes for the **A.** CtBP1 downregulated miRNAs; or **B.** CtBP1 upregulated miRNAs were determined using miRecords data base. Pie charts show GO analysis of predicted target genes using GO Panther. Four top processes over-represented were drilled down to smaller categories.

Using miRecords data base, we obtained 77 predicted miRNAs target genes up- and 30 genes down-regulated by this set of 42 differentially expressed miRNA (Supplemental Table 2). Functional GO analysis of all these genes revealed an enrichment of localization, metabolic processes, cellular process and biological regulation categories, among other biological functions (Figure 5). Interestingly, examining processes within these GO functions; we found important categories overrepresented, such as cell cycle, cell communication, vesicle-mediated transport and primary metabolic process (Figure 5).

Furthermore, we defined validated miRNA target genes using miRTarBase. We found a complete list of 867 miRNAs validated target genes, 430 genes from up- and 437 from down-regulated miRNAs (Supplemental Table 3). GO functional analysis displayed enrichment at metabolic process, cellular process, biological regulation and developmental process categories (Supplemental Figure 1A). Interestingly, analyzing these top four enriched sets, we found again enrichment of genes involved in cell cycle, cell communication and primary metabolic processes (Supplemental Figure 1B).

## DISCUSSION

In this work we generated a MeS experimental mouse model chronically feeding animals with HFD. We found that this diet increased postnatal mammary gland development and proliferation observed by two particular features: high number of branches and TEB; and prominent duct patterns (44% of mice).

Previously it was reported a positive association between number of TEB and breast cancer risk in rodents [23]. It was suggested that this is due to the presence of stem cells in this undifferentiated structure that support mammary gland proliferation and branching [24]. Several studies proposed that early life dietary components, such as HFD, induce the formation of TEB and correlates with increased breast tumorigenesis [25–29]. The increase in proliferation of ductal tree that augment mammographic density is consider a risk factor for breast cancer in humans [30].

In this work HFD induced prominent ducts formation in the mammary glands. The prominent duct pattern was first described by Wolfe who associated this feature with increased breast cancer risk in women [31]. More important, we found that the prominent ducts showed increased expression levels of CtBP1 and Cyclin D1 proteins.

**Figure 6:**
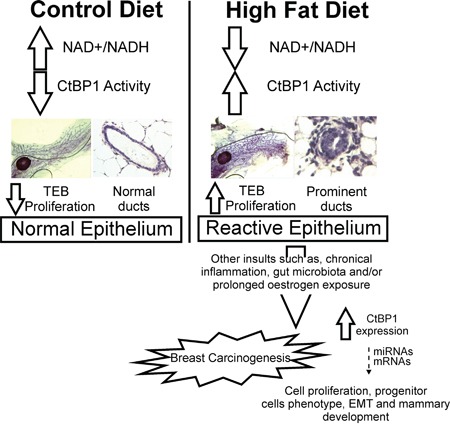
Model proposed for CtBP1 in breast carcinogenesis induced by MeS HFD induces NADH activating CtBP1 expression in the mammary gland which, in turn, induce prominent epithelia ducts and trigger TEB formation. After tumor growth initiates, high CtBP1 expression might provide a worse prognosis to the patients modulating the expression of genes and miRNAs that are involved in cell proliferation, progenitor cells phenotype, EMT and mammary development in breast tumors.

We previously found that CtBP1 depletion impairs prostate tumor growth in mice with MeS [13]. Although, that report demonstrated that CtBP1 is crucial for prostate tumor growth; it was unsuccessful to determine CtBP1 role in prostate carcinogenesis. Here, using breast cancer model we found two significant differences with prostate cancer. First, MeS induces mammary glands proliferation triggering breast carcinogenesis. Second, CtBP1 depletion dramatically decreases breast orthotopic tumor growth in these mice independent of diet. We speculate that HFD induces NADH activating CtBP1 expression in the mammary gland which, in turn, induce prominent epithelia ducts and trigger TEB formation. After tumor growth initiates, high CtBP1 expression might provide a worse prognosis to the patients. Future studies should be performed to determine CtBP1 role in breast tumor progression (See hypothetical model at Figure 6). Hence, we propose to NADH/CtBP1 as a metabolic linker between HFD and breast carcinogenesis.

Importantly, xenograft gene expression analysis determined that CtBP1 significantly increased tumor growth in mice with MeS regulating expression of genes involved in proliferation, stem cell phenotype, EMT and breast development. Previous preclinical and clinical studies demonstrated that CtBP1 overexpression associates with poor breast cancer prognostic [17, 18]. Our work improves this finding showing that CtBP1 might confer a worse outcome for patients with breast cancer associated to MeS.

miRNAs function as master regulators of a wide range of cellular processes by modulating gene expression. Here, we identified 42 miRNAs differentially expressed in CtBP1 depleted xenografts grown in MeS mice that could be promising biomarkers or therapeutic targets (Table 1). Numerous miRNAs from this list were previously found altered in cancer, such as hsa-let-7e-3p, hsa-miR-4448, hsa-miR-223-3p, hsa-miR-3151-5p, hsa-miR-940, hsa-miR-378a-3p and hsa-miR-146a-5p. Previously, it was reported that CtBP1 regulates miR-124 in prostate cancer [32]; however, this is the first report showing that CtBP1 regulates multiple miRNAs in breast cancer. Moreover, GO analysis of miRNA target genes demonstrated that CtBP1 regulates miRNAs associated to metabolism and processes linked to cell communication and cell cycle. Thus, future studies using one particular or multiple miRNAs might be carried out to understand the mechanism of action and future applications of these molecules.

Some recent studies suggest that gastrointestinal tract microbiota modulates cancer development in distant non-intestinal tissues [33]. In addition, HFD has been shown to alter gut microbial communities in both rodents [34] [35] and human beings [36]. Lakritz et al demonstrated that host neutrophil-associated immune responses to intestinal tract microbes significantly impact cancer progression in distal tissues such as mammary glands, and identified gut microbes as novel targets for extra-intestinal cancer therapy [33]. Furthermore, it was demonstrated that postmenopausal women with breast cancer have altered composition and estrogen-independent low diversity of their gut microbiota [37]. Hence, gut microbiota analysis from HFD fed mice should be studied to determine its influence over breast carcinogenesis.

In summary, we identified CtBP1 as a new molecular link that associates MeS and breast cancer. CtBP1 expression in breast cancer tumors might be a powerful tool for diagnosis, prognosis and therapy in a subgroup of breast cancer patients with MeS.

## MATERIALS AND METHODS

### Cell culture, plasmids and transfections

MCF7 (ATCC: HTB-22), T-47D (ATCC: HTB-133), MDA-MB-231 (ATCC: HTB-26), MDA-MB-453 (ATCC: HTB-131), MDA-MB-468 (ATCC: HTB-132), BT-474 (ATCC: HTB-20) cell lines and its derivatives were grown in DMEM medium (GIBCO) supplemented with 10% of fetal bovine serum (FBS) and antibiotics. NIH/3T3 (ATCC: CRL-1658) cell line was grown in DMEM medium (GIBCO) supplemented with 10% of calf serum and antibiotics.

MDA-MB-231 shRNA Scramble and MDA-MB-231 shRNA CtBP1 stable expressing cell lines were generated by lentiviral transduction as previously described [13]. Stable transfected cells were selected with 2 μg/ml puromycin (Sigma-Aldrich) during 10 days and then maintained with puromycin (1 μg/ml).

MDA-MB-231 pcDNA3 cells and pcDNA3 CtBP1 cells were generated by transient transfection using 6 μg of plasmid and polyethylenimine methodology (PEI - PolySciences INC) with PEI:DNA ratio 2:1.

pcDNA3 plasmid was from Invitrogen. pcDNA3 CtBP1 plasmid was kindly provided by Dr. Richard H. Goodman (Vollum Institute, Oregon Health & Sciences University Portland). pGIPZ shRNA Scramble plasmid was from Open Biosystems. shRNA CtBP1 plasmids mix was from Santa Cruz Biotechnology Inc.

LM38-LP cell line, derived from a murine mammary papillary adenocarcinoma [38], were grown in DMEM/F12 medium with non-essential amino acids and 2 μM L-glutamine (Gibco), supplemented with 10% FBS (Internegocios) and 80 mg/ml gentamicin at 37°C.

### Western blot (WB)

Cells were lysed and immunoblotted as previously described [39] using specific antibodies: anti-CtBP1 (621042, BD Transduction Laboratories), anti-β-Actin (4967, Cell Signaling Technology), anti-Lamin A/C (Santa Cruz Biotechnology Inc.).

### RNA isolation, cDNA synthesis and qPCR (RT-qPCR)

RNA was isolated using Tri Reagent (Genbiotech, Buenos Aires, Argentina). cDNA was synthesized from RNA (2 μg) using RevertAid First Strand (ThermoScientific). qPCR was performed as previously described [13] using Taq polymerase (Embiotec, Buenos Aires, Argentina) in a Biorad CFX (Biorad). Data was normalized to β-actin and control. Primer sequences are shown in Supplemental Table 1.

### Cell viability and cell cycle analysis

Cell viability was assayed by MTS (Cell-Titer-96-wells Aqueous non-Radioactive Cell-Proliferation Assay, Promega) according to manufacturer instructions [39]. Cells were exposed to the indicated treatments and after 48 h were stained with propidium iodide (PI) and analyzed by fluorescence activated cell sorting (FACS) as previously described [40].

### Orthotopic xenograft and MeS murine models

Four weeks old female *nu*/*nu* mice (N=24), were housed under pathogen free conditions following the IBYME's animal care guidelines. Mice were randomized into 2 dietary groups and fed *ad libitum* during 16 weeks with control diet (CD; 4,640 kcal/kg, 5% fat) or high fat diet (HFD; 6,040 kcal/kg, 37% fat) as previously described [13]. For HFD, the regular chow food mouse was supplemented with 37% of bovine fat first juice (Fatty, Buenos Aires, Argentina). Body weight was monitored once a week. After 10 weeks mice were randomly distributed into 2 groups and injected in the mammary fat pad (MFP) with MDA-MB-231 shRNA Scramble or shRNA CtBP1 cells (4,8×10^6^). Tumor volume was determined three times a week and calculated as previously described [39]. Animals were sacrificed in the 16^th^ week and tumor, contralateral breast, liver, kidney and blood samples were collected. Mice serum glucose, cholesterol and triglycerides levels were determined as previously described [13]. Histological analysis and IHC studies were performed in 5 μm tissue sections using hematoxilin and eosin (H&E) or specific antibodies.

### Mammosphere formation and clonogenic assays

Four weeks old female *nu*/*nu* mice (N=24), were housed under pathogen free conditions following the University of Buenos Aires's animal care guidelines. Mice were randomized into 2 dietary groups and fed during 16 weeks with CD (2,900 kcal/kg, 5% fat) or HFD (4,450 kcal/kg, 30% fat) as previously described [13]. Then animals were sacrificed, blood samples were extracted by heart puncture and serum was separated. For primary mammosphere formation assay, LM38-LP cells were exposed 144 h to 2.5% of CD or HFD serum (CDS or HFDS). Then 10^4^ cells were seeded in low attachment plates and grown in DMEM-F12 medium (Gibco) supplemented with B27 (1:50) (Life Technologies) and 20 ng/ml EGF (BD Biosciences) for 7 days and mammosphere number and diameter were determined as previously described [41].

For secondary mammosphere formation assay, LM38-LP-derived mammospheres were treated with 2.5% of CDS or HFDS for 144 h. Primary mammospheres were enzymatically dissociated with 0.05% of trypsin for 15 min at 37°C and cell suspension was used to generate a second mammosphere assay [41].

Clonogenic assay was performed as previously [42] with 3×10^3^ cells treated with CDS or HFDS for 6 days.

### Mammary whole mount

Mammary whole mount was performed as previously [43]. Briefly, fourth inguinal MFP were whole mounted in a slide, fixed with Carnoy, hydrated and stained with aluminum carmine red solution. Samples were dehydrated and cleared with xylene. Number of branches, lateral branches and terminal end buds (TEB) were quantified from pictures with x10 magnification of each mammary whole mount using Cell count tool of Image J software.

### miRNA microarrays

GeneChip miRNA 4.0 arrays (Affymetrix) were hybridized with RNA from shRNA Scramble and shRNA CtBP1 MeS xenograft tumors. Data normalization and analysis were performed using Expression Console™ Software 1.3.1 and Affymetrix^®^ Transcriptome Analysis Console (TAC) Software. Differentially expressed miRNAs were identified using ANOVA and fold change (p≤ 0.05).

CtBP1 regulated miRNAs were divided into up- and down-regulated miRNAs. The threshold was set to 1.5 fold change for up-regulated and −1.5 for down-regulated genes. Predicted miRNAs target genes from each group were obtained using miRecords which integrates predictions from 11 different data bases (http://c1.accurascience.com/miRecords/). We only consider as valid targets those genes that were predicted by at least 3 data bases. Duplicated genes between both groups were eliminated.

Furthermore, validated target genes of differentially expressed miRNAs were determined using MiRTarBase (http://mirtarbase.mbc.nctu.edu.tw/). Gene Ontology (GO) analysis was performed using Panther software (http://www.pantherdb.org/).

### Statistical analysis

All results are given as mean and standard deviation (SD) of three independent experiments. Student t tests were used to ascertain statistical significance with a threshold of P < 0.05. For *in vivo* experiments, two-way ANOVA followed by Bonferroni test were performed. Shapiro–Wilk and Levene tests were used to assess normality and homogeneity of variances. *, P < 0.05; **, P < 0.01;***, P < 0.001.

## SUPPLEMENTARY FIGURES AND TABLES






