# Supplementary material for: CtBP1 associates metabolic syndrome and breast carcinogenesis targeting multiple miRNAs
Source: Oncotarget. 2016 Feb 25;7(14):18798–811. doi: 10.18632/oncotarget.7711 (PMC4951330; doi:10.18632/oncotarget.7711)
Supplement: Supplementary file 1 [file oncotarget-07-18798-s001.pdf]

SUPPLEMENTARY FIGURE AND TABLES

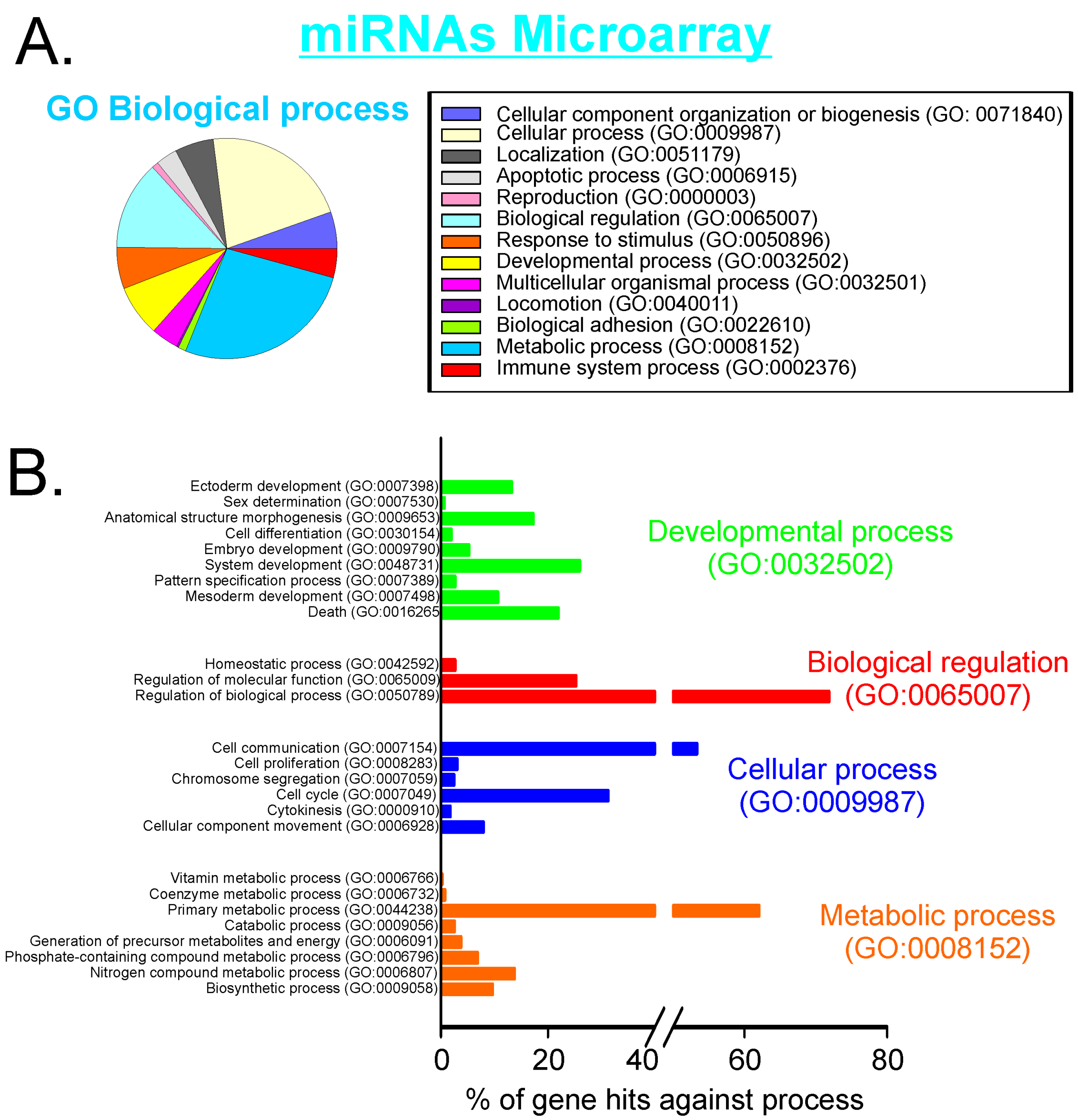

Supplemental Figure S1: CtBP1 modulates miRNAs involved in metabolic process, proliferation, developmental process and cell comunication. GeneChip miRNA 4.0 Affymetrix was hybridized to total RNA from xenografts with CtBP1 knockdown or control grown in mice with MeS. Validated target genes were determined using miRTarBase. **A.** Pie charts show GO analysis using GO Panther. **B.** Four top processes overrepresented were drilled down to smaller categories.

Supplemental Table S1: Sequences of specific primers used for RT-qPCR experiments

Gene	Accession	Primer Forward Sequence	Primer Reverse Sequence
Cyclin D1	NM_053056	GCGGAGGAGAACAAACAGAT	TGAGGCGGTAGTAGGACAGG
E-cadherin	NM_004360	AAGGTTCACCCAGCACCTTGCA	GGCAGAGGGACACACCAGTGTAGTAA
CtBP1	NM_001328	TACAGCGAGCAGGCATCC	TGGTCCTTGTTGACACAGTTC
b-Actin	NM_001101	AAGATCATTGCTCCTCCTGAGC	CATACTCCTGCTTGCTGATCCA
Gli1	NM_001167609	CACTGGTCTGTCCACTCTTCG	GCTGCTGCGGCGTTCAAG
RIP140	NM_003489	GCTGGGCATAATGAAGAGGA	CAAAGAGGCCAGTAATGTGCTATC
Vimentin	NM_003380	CACTCCCTCTGGTTGATAC	GTGATGCTGAGAAGTTTCG
Slug	NM_3068	TCGGACCCACACATTACC	CAGATGAGCCCTCAGATTTG
Snail	NM_005985	CCTGCGTCTGCGGAACCTG	GTTGGAGCGGTCAGCGAAGG
RANK	NM_003839	GCTACTTCTCTGATGCCTTTTCC	CCCATGATGTTCTACTCTCTTTCC
OPG	NM_002546	GAAGGGCGCTACCTTGAGAT	GCAAACTGTATTTCGCTCTGG

Supplemental Table 2: List of predicted target genes for miRNA upregulated or downregulated by CtBP1 according miRecords data base

See supplementary file 1

Supplemental Table 3: List of validated target genes for miRNA upregulated and down regulated by CtBP1 according MiRTarBase data base

See supplementary file 2
