# Supplementary material for: CtBP1 associates metabolic syndrome and breast carcinogenesis targeting multiple miRNAs
Source: Oncotarget. 2016 Feb 25;7(14):18798–811. doi: 10.18632/oncotarget.7711 (PMC4951330; doi:10.18632/oncotarget.7711)
Supplement: Supplementary file 2 [file oncotarget-07-18798-s002.docx]

***Supplemental Table 2. List of predicted target genes for miRNA upregulated or downregulated by CtBP1 according miRecords data base***

| **miRecords** | |
| --- | --- |
| **CtBP1 upregulated miRNAs** | **CtBP1 downregulated miRNAs** |
| CREB3L2 | IGF1R |
| LONRF2 | SMAD5 |
| IYD | C18orf25 |
| FREM2 | ENAH |
| MKNK2 | KCNMA1 |
| NANOS1 | LOC203547 |
| N4BP1 | AGPAT3 |
| HIF3A | FOXN3 |
| GREB1 | PRKCA |
| WHSC1 | SEC62 |
| AMOTL1 | UVRAG |
| LRRC15 | ZNF264 |
| TP53RK | ARHGAP29 |
| SSH2 | ADAMTS5 |
| PCDH21 | FZD4 |
| PCGF5 | SHANK2 |
| C1orf21 | RALGPS1 |
| DHDDS | HELZ |
| SH3TC2 | PSD3 |
| ZFYVE20 | NT5DC3 |
| ADCY1 | RNF125 |
| MTMR3 | RAB22A |
| GPR107 | PHC3 |
| NLGN2 | PGAP1 |
| PCBP4 | JHDM1D |
| KIAA1219 | PURB |
| SCN3B | OTUD4 |
| HIF1AN | DCP2 |
| HEMK1 | KSR2 |
| ST8SIA3 | MOBKL1A |
| BRPF3 |  |
| KIF1B |  |
| GIT2 |  |
| SPOCK2 |  |
| RIMS3 |  |
| KIAA0247 |  |
| TFCP2L1 |  |
| NEK6 |  |
| TRAM2 |  |
| KLF12 |  |
| CA5B |  |
| SNTB2 |  |
| NFAT5 |  |
| FUT9 |  |
| PCGF3 |  |
| SLCO2A1 |  |
| HDLBP |  |
| SCN2B |  |
| H6PD |  |
| AKAP6 |  |
| RNF8 |  |
| PRPF4B |  |
| RNMT |  |
| JRK |  |
| VAPA |  |
| KPNA1 |  |
| ZNF621 |  |
| ENTPD1 |  |
| MRAS |  |
| ZBTB40 |  |
| SRGAP2 |  |
| XRN1 |  |
| NSL1 |  |
| GK5 |  |
| TRIOBP |  |
| RBM9 |  |
| COX19 |  |
| ADAR |  |
| CUGBP2 |  |
| RAB3IP |  |
| FIGNL2 |  |
| EDA |  |
| BMF |  |
| RECQL5 |  |
| PTGIS |  |
| DCX |  |
| SGCD |  |
