# Supplementary material for: CtBP1 associates metabolic syndrome and breast carcinogenesis targeting multiple miRNAs
Source: Oncotarget. 2016 Feb 25;7(14):18798–811. doi: 10.18632/oncotarget.7711 (PMC4951330; doi:10.18632/oncotarget.7711)
Supplement: Supplementary file 3 [file oncotarget-07-18798-s003.docx]

***Supplemental Table 3. List of validated target genes for miRNA upregulated and down regulated by CtBP1 according MiRTarBase data base.***

| **MirTarBase** | | | | | |  | |  | |  | | | |  | |  |  |
| --- | --- | --- | --- | --- | --- | --- | --- | --- | --- | --- | --- | --- | --- | --- | --- | --- | --- |
| **CtBP1 upregulated miRNAs** | **CtBP1 upregulated miRNAs** | **CtBP1 upregulated miRNAs** | **CtBP1 upregulated miRNAs** | **CtBP1 upregulated miRNAs** | **CtBP1 upregulated miRNAs** | | **CtBP1 downregulated miRNAs** | | **CtBP1 downregulated miRNAs** | | **CtBP1 downregulated miRNAs** | **CtBP1 downregulated miRNAs** | **CtBP1 downregulated miRNAs** | | **CtBP1 downregulated miRNAs** |  |  |
| HMGA2 | LHFPL2 | MTFR1 | STARD7 | IRAK1 | CD86 | | EP300 | | MGST3 | | TPP1 | BRIP1 | CCND2 | | TSEN2 |  |  |
| EIF3J | ARCN1 | IRS2 | PDP2 | ROCK1 | IFIT5 | | CDH2 | | NCOA6 | | AARS2 | UHMK1 | REEP3 | | HSPA1B |  |  |
| SMC1A | COX1 | IRS4 | CLTC | BRCA2 | C1orf21 | | RAC1 | | RNF19A | | CHAF1A | MTL5 | LMNB2 | | CPT2 |  |  |
| WNT1 | SLC12A4 | PPP1R10 | TERF1 | BRCA1 | GIMAP4 | | HBEGF | | DPH3 | | PARP1 | ZNF646 | KIF20A | | NLGN2 |  |  |
| CCND1 | AEBP2 | ZNF284 | PAX3 | FAF1 | CCR9 | | IGF1R | | GALNT1 | | DESI1 | AQR | ZNF485 | | MLX |  |  |
| MPL | PET112 | YWHAE | CCDC97 | CCNA2 | SAMD9L | | PTPN12 | | CSNK1A1 | | CYP3A5 | CLIP1 | VTA1 | | SOD1 |  |  |
| RABGAP1L | UROC1 | SCD | HLA-C | PA2G4 | IFIT3 | | PTPN13 | | CNNM3 | | AHSA1 | PPARGC1A | RAP2B | | PNISR |  |  |
| DAD1 | IGF1R | FDPS | GTPBP8 | SNAP25 | CD80 | | ITGA9 | | LFNG | | SLC16A6 | IMPDH2 | NA | | PHACTR2 |  |  |
| MYCN | BSDC1 | KRBOX4 | VGLL4 | IL8 | SPP1 | | SOCS2 | | KIAA1147 | | PALM2-AKAP2 | AKAP8L | BRAF | | ASB16-AS1 |  |  |
| WDR67 | PTK2 | BRI3 | ANKRD40 | NFKB1 | SLPI | | DNMT3A | | GPR107 | | CDC42SE2 | YWHAB | SUFU | | SEC31A |  |  |
| FAM219B | CDC5L | CHD7 | HNRNPUL1 | CDKN1A | L1CAM | | SOX5 | | ANKRD40 | | XPR1 | C1QBP | VEGFA | | TTC28 |  |  |
| C11orf91 | EN2 | PYCR1 | ND1 | EGFR | MSC | | ACVR2B | | ATP9A | | ATRX | TPR | TUSC2 | | PML |  |  |
| CENPP | SSB | SMAP2 | DSP | MTA2 | TUBB4B | | TLN2 | | ASH1L | | KPNA1 | HNRNPDL | NPNT | | CCND2 |  |  |
| TMEM107 | SLCO3A1 | WDFY3 | UBN2 | CD40LG | CHAMP1 | | BMI1 | | NDUFC2 | | USP46 | CACTIN | GALNT7 | | PTMA |  |  |
| SREBF1 | RRAGC | CRK | ZNF805 | FAS | EIF6 | | SRCAP | | ANKRD17 | | C1orf198 | CMTR2 | MYC | | ECH1 |  |  |
| DAAM1 | ZNF236 | SRSF2 | RBBP4 | CDKN3 | MBNL1 | | SLC16A9 | | IQGAP1 | | NFATC2IP | ACTN4 | TOB2 | | AGK |  |  |
| DGCR8 | SEMA4B | PGD | CBX5 | KIF22 | CALR | | HNRNPA1 | | STX3 | | PPT1 | CHTF18 | MSC | | STARD7 |  |  |
| RAP1A | SERBP1 | RMND5A | AMPD2 | ERBB4 | TRIM5 | | AGO1 | | SKA2 | | ZNF805 | DYM | CYP19A1 | | ACSL4 |  |  |
| RPA1 | XIAP | ZNF652 | MKI67 | SMAD4 | IL6ST | | ELF1 | | FOXM1 | | DHPS | PRMT1 | GRB2 | | E2F5 |  |  |
| SKIV2L | GTF2I | PSMA6 | SUB1 | TLR4 | GNAI2 | | HIST2H3A | | LPCAT1 | | PPP1R15B | SUFU | VPS37B | | DVL1 |  |  |
| AGO1 | JMJD1C | SLC38A2 | RSL1D1 | WASF2 | MGEA5 | | TYMS | | YWHAE | | DDIT4 | FYCO1 | DDX39A | | PSMA3 |  |  |
| RPS27 | OTUD5 | LRRC41 | SEC23IP | NFIX | MKI67 | | ZNF254 | | ZNF12 | | CUX1 | ARC | C3orf38 | | EIF4H |  |  |
| ARNT2 | NOLC1 | RANBP2 | RNMT | IRF7 | COL1A2 | | HN1 | | SPIRE1 | | CDA | NFE2L1 | GMEB2 | | ACAP2 |  |  |
| GPM6B | PPIG | SVIL | SGSM3 | RAD54L | TRIO | | LANCL1 | | STAT3 | | PRR14L | BAG6 | DCLRE1B | | OCRL |  |  |
| TTLL12 | PSMD2 | CCNG1 | ZFP3 | PPP1R1C | FAM208A | | SETD1B | | ARL6IP1 | | CHGB | SDF4 | LAMP1 | | MRPL37 |  |  |
| HIST2H2BF | UGGT1 | ZNF256 | GLUL | TRIM14 | LY6K | | SLC4A7 | | PGM2L1 | | LRPPRC | SART3 | CMTM6 | | FOXC1 |  |  |
| CELF2 | VAMP2 | COPG1 | POLD1 | TMSB15A | TMEM131 | | RBBP4 | | ETNK1 | | MPP5 | GDE1 | ZBTB14 | | TIGD1 |  |  |
| AGO2 | LSR | PDCD11 | WDR4 | ITGB2 | ZAK | | ACACA | | IDI1 | | ALG9 | SYDE2 | CHAMP1 | | CSTF3 |  |  |
| VPS13D | KIAA0355 | CNBP | PPP1R2 | SNTG2 | AES | | PPP6C | | H2AFV | | TNFRSF1B | SLC16A9 | IGF2BP1 | | FOXRED2 |  |  |
| RPL10 | RPSA | USP37 | RAI2 | RSAD2 | ELMSAN1 | | USP53 | | SLC35F5 | | PPIA | YEATS2 | SREBF1 | | NME4 |  |  |
| SPCS2 | DCAF6 | UBE2V2 | PLCXD1 | TIMELESS | AXL | | UHMK1 | | C1D | | EPDR1 | AP4B1 | TRAF1 | | PTBP1 |  |  |
| DCAF8 | UHRF1BP1 | UBE2H | UTP15 | PRR15 | CTNNA1 | | CHD7 | | TJP1 | | LUC7L3 | ING3 | B4GALNT3 | | KPNA6 |  |  |
| CTC1 | OTUB1 | LMNA | THADA | PEX11G | LIF | | KDM3A | | CAND1 | | VCL | SET | KLF10 | | SLC22A23 |  |  |
| NAA60 | PIGP | STAT3 | ZNF451 | PLEKHA4 | AMPD2 | | MED6 | | DNAJC10 | | BRCA2 | EIF4A1 | ZNF100 | | OXSR1 |  |  |
| PHF3 | LMLN | TRPV1 | CREBBP | BGLAP | PTPN13 | | RPS6KB1 | | PTPN14 | | SP3 | KXD1 | EEF1B2 | | RTN3 |  |  |
| TUBA1B | PPP2R1A | MATR3 | ZNF770 | SYT12 | AZIN1 | | LIN7C | | NUP210 | | TRIP11 | TUSC2 | USMG5 | | KLHL15 |  |  |
| SHANK1 | HIPK1 | HNRNPC | CLDN4 | STAT1 | SLC35F5 | | GTF2E1 | | GNA13 | | TOB1 | FAM115A | ALDOB | | HSP90AA1 |  |  |
| SKA2 | SERF2 | HS6ST2 | ZKSCAN7 | LMO2 | PHF3 | | KHDRBS1 | | ELK4 | | FZD6 | POLR2F | TRAFD1 | | BYSL |  |  |
| SPTBN1 | RBM4 | WDR48 | NICN1 | CD83 | FAM127B | | WDR37 | | RAD23B | | GNG12 | PSMD3 | BRWD3 | | VPS13C |  |  |
| ND2 | RBM14 | RBM8A | RPLP2 | IFIT1 | MYH9 | | ZNF17 | | PTPLB | | KLF9 | DMXL2 | RBAK | | BLOC1S3 |  |  |
| MED13L | HMGB1 | SCYL1 | DCBLD2 | IFITM1 | RNF103 | | SCARB2 | | WASL | | ZNF148 | PRRC2A | TMEM199 | | RPS7 |  |  |
| RCOR3 | GMPS | C19orf48 | IRGQ | OLFML2A | UAP1 | | TMEM70 | | HIPK2 | | FAM46C | COLGALT1 | DHX15 | | NOLC1 |  |  |
| MACF1 | DIAPH1 | FOXD4L6 | CA5B | ISG15 | G3BP1 | | CCNI | | FGF20 | | SIAE | KCNJ3 | TCP1 | | YWHAZ |  |  |
| RDX | STAM | PIGN | COX14 | UHRF1 | TSC1 | | TNRC6A | | CHD1 | | PITHD1 | HSPA1B | SLC25A2 | | MLF1 |  |  |
| PCBP2 | YWHAQ | SPCS3 | PSD3 | IFITM3 | FAM120A | | VEZF1 | | TMPPE | | MTUS1 | EIF3E | KIAA1468 | | STXBP1 |  |  |
| UBAP2L | APPL1 | TNFAIP1 | RPL27A | RGS13 | SP7 | | CCDC88C | | TIPARP | | SAMD8 | HSPA1B | AGO1 | | STXBP3 |  |  |
| CDCA3 | MARCH5 | MS4A10 | CWC15 | CCL8 |  | | SEC24B | | ST3GAL2 | | SLC25A37 | RAN | ZNF239 | | WNK1 |  |  |
| CABLES1 | SQLE | MSMO1 | NT5DC2 | MTUS2 |  | | MCUR1 | | SLC4A7 | | TRIM8 | FIP1L1 | NDE1 | | FLNA |  |  |
| BTRC | TIMM50 | ARID1A | OR7D2 | BLMH |  | | TCF20 | | C21orf58 | | RORB | LDLR | ID2 | | FAM118A |  |  |
| C12orf49 | NDUFA3 | NCKIPSD | HIF1AN | MCPH1 |  | | SOCS5 | | LPCAT4 | | HSPA4 | SMG7 | CASC3 | | RABEP2 |  |  |
| TIMP3 | NDUFS5 | NCBP1 | NUDT15 | NMI |  | | TBC1D14 | | GCNT1 | | ZNFX1 | IGSF3 | ZNF473 | | RNPS1 |  |  |
| WBSCR16 | NRSN2 | COX16 | CDH18 | HSPA1A |  | | PPP2R2A | | C7orf43 | | CALM1 | ZNF281 | NDFIP2 | | LDHA |  |  |
| SLC2A11 | SALL1 | NUDT8 | FAM160B1 | TRIM22 |  | | RGMB | | ZNF485 | | TMEM59 | USP24 | CPNE1 | | CEP164 |  |  |
| NF1 | COX3 | PHKA1 | DZIP1 | ITGBL1 |  | | BNIP2 | | ZBTB43 | | STAT5A | TIPRL | ERRFI1 | |  |  |  |
| LRRC8A | RABL6 | TXNRD1 | TDRD7 | SPATS2L |  | | NFE2L2 | | TAX1BP1 | | NUCKS1 | RNGTT | TRA2B | |  |  |  |
| RUNX1T1 | SUPT4H1 | ELMSAN1 | TCF4 | POLE2 |  | | MCF2L | | RFX7 | | ZBTB38 | MOGS | HIST1H3H | |  |  |  |
| DTNB | PACS2 | CTSA | ENSA | IFI44 |  | | EFHD1 | | SNX17 | | P4HA1 | ARNTL2 | TXNIP | |  |  |  |
| SCMH1 | MTMR14 | PRRC2A | GTF3C1 | OASL |  | | HIC2 | | DCAF6 | | TACC1 | ZBTB9 | KDM5C | |  |  |  |
| YWHAG | LUZP1 | SPAG9 | TNFRSF1A | LTB |  | | SAE1 | | KANK1 | | PINK1 | UBA1 | ZNHIT3 | |  |  |  |
| RHBDD2 | PFAS | AGMAT | CCDC113 | PBLD |  | | RPS6KA5 | | CNEP1R1 | | ESYT1 | GPBP1L1 | BPTF | |  |  |  |
| ND5 | SUZ12 | NCKAP5L | MYO9B | IFI44L |  | | TRIM36 | | MT-CO1 | | PDPR | XPO6 | CPSF7 | |  |  |  |
| NDST1 | PAPD4 | ZFP62 | COX10 | PDIK1L |  | | SYNCRIP | | TNRC6B | | DDI2 | CLCN4 | EDARADD | |  |  |  |
| IGF2BP3 | QSOX1 | EIF4EBP2 | PRAMEF13 | MR1 |  | | PWWP2A | | TMEM107 | | PELI1 | NA | CHCHD4 | |  |  |  |
| EIF4A1 | SPATA13 | POLR3D | PMPCA | IFI27 |  | | BLCAP | | CUL4B | | KIAA0754 | HSPA1A | DCTN4 | |  |  |  |
| BAHCC1 | DHX57 | PRPF8 | MRPS2 | TBP |  | | MTMR14 | | ZKSCAN1 | | SLK | IARS2 | KREMEN1 | |  |  |  |
| CARM1 | KATNB1 | ATP6 | RACGAP1 | ELL3 |  | | CKS1B | | TFAM | | CYB561A3 | NECAP2 | GOLGA8B | |  |  |  |
| DYRK2 | COL6A1 | ZFAND5 | CCDC106 | METTL7A |  | | CLK1 | | MTDH | | HSPA1A | NFX1 | ENO1 | |  |  |  |
| ND3 | DHX15 | BAZ1B | CYP2B6 | MCM10 |  | | LMO4 | | SLC39A14 | | CHCHD10 | CAMKK2 | NDFIP1 | |  |  |  |
| PIGS | MTCH2 | KATNAL1 | CDKAL1 | LINC00304 |  | | VKORC1L1 | | RAB5B | | IQSEC1 | LBR | FNDC3B | |  |  |  |
| ALG13 | KIAA0100 | BMP2K | PSME4 | SDCBP2 |  | | GOLPH3 | | ASXL2 | | CHD2 | RIOK1 | DYRK2 | |  |  |  |
| RPN2 | SUGP2 | SPN | SETD1A | STON2 |  | | ZNF248 | | ENTPD7 | | BAG6 | KIF13B | HIC2 | |  |  |  |
| RPL12 | NCLN | PA2G4 | CXCR4 | BCL2A1 |  | | RANBP6 | | INADL | | NT5DC3 | HACE1 | MXD4 | |  |  |  |
| NME4 | ATXN2L | RPRD2 | KIT | COL13A1 |  | | PRDM4 | | SMCO4 | | MYO1B | PTOV1 | UBA52 | |  |  |  |
| IVD | CHD9 | TUBB4B | CFH | IRF5 |  | | AHNAK | | ZCCHC10 | | UBR4 | ETF1 | HIST1H2BD | |  |  |  |
| JAZF1 | PES1 | TRRAP | IRAK2 | MX2 |  | | WDR43 | | SMAD7 | | EIF3D | FMR1 | CYB5R3 | |  |  |  |
| ND4 | NUP155 | SLCO4A1 | TLR2 | VWCE |  | | MCCC2 | | PLSCR1 | | ETNK1 | DNAJC14 | PCCB | |  |  |  |
| VARS | GNG5 | CCRN4L | FADD | EPSTI1 |  | | IL6ST | | TNRC6A | | TALDO1 | TTC17 | ACTB | |  |  |  |
| RNF26 | ARHGAP19 | USE1 | TRAF6 | OSBPL1A |  | | ARCN1 | | IGSF3 | | USP22 | EEA1 | KLF14 | |  |  |  |
